# Correspondence

**Published:** 1897-09

**Authors:** John J. Repp

**Affiliations:** University of Pennsylvania


### CORRESPONDENCE.

EDITORS JOURNAL OF COMPARATIVE MEDICINE, ETC.:

It seems to me that one of the requisites for the attainment of that high standing which all devotees of the profession of veterinary medicine desire for it is a uniformity of the degrees conferred by the schools.

It is doubtless of value to the practitioner of human medicine that almost everyone knows the significance of the M.D. which the graduates in that profession are permitted to attach to their names. The graduates in veterinary medicine should be accorded a similar benefit.

At this time there are in America almost as many varieties of degrees as there are colleges. Only the specialist knows the import of the letters which are appended to the name of the graduate of veterinary medicine. This is unfortunate.

It is not my purpose to enter into a discussion of ways and means, but to leave the matter with the profession, begging leave to suggest that it would be a fit topic for consideration at the meeting of the Association at Nashville in September.

Yours sincerely,

JOHN J. REPP.

University of Pennsylvania.

